# Organising Research and Development for evidence-informed health care: some universal characteristics and a case study from the UK

**DOI:** 10.1017/S1744133121000074

**Published:** 2021-10

**Authors:** Anthony J. Culyer, Kalipso Chalkidou

**Affiliations:** University of York – Centre for Health Economics, York, United Kingdom of Great Britain and Northern Ireland

**Keywords:** Characteristics of health care R&D, public goods, R&D in health care, R&D in UK, supplier-induced demand

## Abstract

Research and Development (R&D) in health and health care has several intriguing characteristics which, separately and in combination, have significant implications for the ways in which it is organised, funded and managed. We review the characteristics, some of which apply under most circumstances and others of which may be context-specific, explore their implications for the organisation and management of health-related R&D, and illustrate the main features from the UK experience in the 1990s.

## 1. Introduction

Research and Development (R&D) in health and health care has several intriguing characteristics which, separately and in combination, have significant implications for the ways in which it is organised, funded and managed. This paper takes a broad approach by identifying eight significant aspects that usually characterise health care R&D (and other fields of R&D), exploring them in principle and then applying them to an analysis of some historic changes in the way R&D was managed in the UK's National Health Service (NHS). The UK had its own history of health care, of course, and our aim is to provide a widely applicable way of thinking about Universal Health Coverage (UHC) and the public organisation of R&D in support of it, in low-, middle- and high-income countries. The NHS case study is not offered as a model of ‘how to do it’ but as an example demonstrating that having a rational managed and targeted R&D strategy is actually possible.

Our approach is largely informed by economics, which clarified several technical but important aspects of public choice in the 1950s and 1960s. The relevant economic elements are explored here in a way that is intended to be comprehensible and useful to the whole of the health research community, whatever their interest and whatever their disciplinary background. The context considered is one in which a large proportion of health care (though not necessarily health care R&D) is funded from, and perhaps provided by, public agencies, and the health care system can be broadly considered to be, or aspire to be, both comprehensive in terms of the benefits offered and universal in terms of population coverage (Culyer and Chalkidou, 2019; Hauck *et al*., 2019). What will be achievable in either of these dimensions clearly depends on the general state of development of the economy and whether it has disposed itself in a purposeful way. However, all countries face constraints that limit ambition both for the health care system itself and for R&D. Good system design and making the best use of the resources available are always priorities, whatever a country's wealth and whatever its history and culture – but the wealth, the history and the culture will help to define what ‘good system design’ is and imply that the design cannot be independent of the economic and cultural context (Culyer, 2010; Chalkidou *et al*., 2017; Li *et al*., 2017; Culyer *et al*., 2018; Norman *et al*., 2018; Isaranuwatchai *et al*., 2019).

Since the work of the international Commission on Health Research for Development in the late 1980s (Commission on Health Research for Development, 1990), there have been several international initiatives, convenings and resolutions calling for health systems around the world to recognise the importance of and invest in research for health (Abbasi, 2004; Cohred; Forum, 2015). The most recent of these is the R&D Accelerator (WHO) (one of seven such ‘accelerators’ meant to accelerate progress towards the Sustainable Development Goals), managed by the WHO and (in the case of R&D) supported by the Wellcome Trust. The Accelerator has four main thrusts: to identify best practices for research and pipeline coordination, building innovation hubs and other routes for generating new health-improving knowledge; to ensure that promising innovations reach those who need them, working in partnerships with governments, funders and the private sector; to remove barriers to innovation and scaling up; and to explore how to shift priorities for research and innovation to the country level [(particularly in low- and middle-income countries (LMICs)]. In its recommendations, the Accelerator Advisory Group, like similar groups before it, called for countries to develop a national health research agenda and for WHO to coordinate, support and advise countries on how to prioritise their research needs. Meanwhile, the emergence of Health Technology Assessment institutions in LMICs has proved a significant energiser of research, which has already led to the creation in some countries of well-integrated structures, e.g. Thailand (HITAP) and Brazil (CONITEC), with others currently being set up, such as the National HTA agency of India (HTAIn), the Chinese (less transparent perhaps and certainly less well publicised) equivalent of NICE recently launched and the HTA processes considered in the context of South Africa's National Health Insurance Scheme (iDSI, 2018; MacQuilkan *et al*., 2018; National Health Insurance Bill, 2019, sec. 7, sec, 57). These are important steps but they are incomplete. Accelerating relevant R&D for UHC requires much more than the making of lists; it requires also the local development of the mechanisms for making those lists; it cannot be achieved in the style of a ‘command economy’ but requires an understanding of what motivates key players and what R&D is needed at the workface; it is not only top-down but needs also to be bottom-up, requiring, as it does, the collaboration and cooperation of a wide variety of stakeholders, including the clinical professions, researchers in industry and the universities, funders, public sector managers, and strategic thinkers. Creating a system structure that provides the appropriate incentives for all such groups is the most important required innovation of all.

We begin at a fairly general, even abstract, level, on the grounds that some of the characteristics that need to be taken into account are themselves universal and much less dependent on local history and culture than others. Three of the intriguing characteristics were highlighted long ago by Nobel economics prize-winner Kenneth Arrow in 1962 (Arrow, 1972) but we shall add others. We then draw on the experience of England in developing its R&D reforms in the 1990s as illustrations of how one country addressed the many intriguing characteristics of R&D in practical terms and terms that were also consistent with the then current political and institutional features of the health care R&D scene in England. We then describe the concrete institutional capacity building that followed and draw some conclusions about *ways of thinking* about supporting R&D in health and health care that maximise the chances of success in other countries.

The main attributes of health care R&D (like most R&D) considered here are these:

1. Publicness – R&D is a public good;
2. Supplier-induced demand (SID) – R&D has considerable potential for supplier-induced demand (induced, that is, by the suppliers of R&D rather than the suppliers of health services);
3. Informational asymmetries – these are all-pervading;
4. Joint production – there are substantial elements of joint production in the simultaneous delivery of health care and the production of R&D;
5. Fragmentation – funding typically comes from a diversity of sources (private and public) having a diversity of both complementary and competing objectives;
6. Uncertainty – the output and value of R&D is inherently uncertain;
7. Outcomes – the measurement of the outcome of R&D is in its infancy;
8. Managerial motives – health-related R&D is largely undertaken in both for-profit (e.g. industry) and non-profit (e.g. universities) institutions whose motivation and efficiency are poorly understood and whose behaviour is difficult to predict.

Each of these characteristics merits an essay in its own right. Here, the focus is on them as elements that have to be taken into account by governments seeking a rational structure for supporting R&D on a national or regional level that also generates incentives for the various players to enhance its quality and relevance. The paper begins by developing the particular analytical characteristics of R&D in health care common to all systems in all contexts. The next section develops the context-free characteristics and indicates their potential for resource misallocation and, by implication, what measures in the design of a system of support might counter such forces. The next section describes some context-specific major policy changes that occurred in England and which were informed, at least in part, by an analysis of this kind. The final section draws some conclusions: ways of thinking about system design that is as applicable in LMICs pondering a national R&D strategy, agenda and/or budget as they are in high-income countries – always with, of course, due regard to local contextual factors. Our proposals are not, therefore, a substitute for thought. Rather, they offer an approach in which thought might be profitably structured in a context-sensitive way.

## 2. The special characteristics of R&D in health care

There is no clear dividing line between ‘research’ and ‘development’ beyond the fact that development intrinsically demands the application of theory to specific problems and that the purest of research tends to be theory-focused. The balance between the two varies by context. For example, in commercial pharmaceuticals research, the ‘D’ might be seen as beginning with Phase I clinical trials; in non-commercial research into health inequalities, the ‘D’ element is effectively delivered (if at all) by public policy pilots or actual implementation after political modification. The ‘R’ is what goes on before. In practice, the scope of R&D must be a matter for deliberation and policy judgement exercised in a local context. The NHS in the UK provides, as will be seen, an example of such pragmatism.

### 2.1 Publicness

While some R&D undertaken in health care systems is largely private rather than public in character (e.g. that supported by pharmaceutical firms), much else has the characteristic of publicness.

The classic definition of a public good is that by another Nobel economist, Paul Samuelson, in 1954 (Samuelson, 1954, p. 387):

Each individual's consumption of such a good leads to no subtraction from any other individual's consumption.

Street lighting and national defence are frequently cited examples.

A similar definition can be found in a later piece by Samuelson (Samuelson, 1955, p.350):

Each man's consumption of it … is related to the total … by a condition of equality rather of summation.

Thus, a public good, if provided for anyone, is also provided for all (though each may attach a different value to it).

This defines the purely public end of what is often a spectrum or degree of ‘publicness’ and ‘privateness’, in which intermediate points are characterised by some diminution of others' consumption, though by less than the amount consumed by another (as when a public facility becomes congested) or when there are relatively few who value the good highly enough to want to consume it under any circumstances (as when the public good, such as a village clocktower, is essentially local in character). It also makes plain that the character of publicness is a technical characteristic of the good in question rather than the product of the particular arrangements in existence for its production and distribution. It is also the technical character of the demand for the good rather than its ownership that defines publicness. For example, public goods like preventive public health measures could be provided by private agencies provided there was a mechanism for expressing the public's demand for them. The idea that a public good can be defined in terms of an exclusion principle (Musgrave, 1959) is somewhat different, though also demand-based. This defines publicness (sometimes termed a social want) in terms of whether a consumer can be excluded from the benefits of a good if they declined to pay a contribution or price towards it. If not, it would be considered a public good. Samuelson's technical definition is that used here. Whether and how the beneficiaries of a public good contribute to its production is a matter separate from the inherent character of the public good. Likewise, whether it is possible or desirable for the public good to be efficiently supplied by the private sector is a separate issue.

R&D in health care, whether biomedical or applied social science, has the typical characteristics of a public good. Once it is done, it is available to society as a whole at effectively zero additional marginal cost of production and the use of it by one consumer does not diminish what remains for others to use. Its benefits accrue to a wide variety of individuals and institutions: most directly to those who manage and deliver health care but, more ultimately, to the consumers of health care themselves. Although it is possible to establish exchangeable private property rights in the information produced by R&D through intellectual property rights and patents, in general this has not been seen as desirable in the case of much R&D, on the grounds that its production has often been funded at public expense and that it should therefore be freely available, that publication is one of the major mechanisms through which quality control is exercised via peer review, and because future research productivity is very dependent upon what has gone before, much of which will, again, have been publicly funded and generated in public institutions.

Several problems immediately arise out of this publicness of R&D. One is that mechanisms for revealing the collective demand for R&D are required. A second is the creation of mechanisms for valuing and prioritising the R&D projects and programmes to be supported and to determine the appropriate level of resourcing each should have. A third is the determination of the overall resource commitment to health care R&D in general. Crossing all of these issues is a more fundamental one: should the broad character of the mechanisms for solving these problems be a central mechanism, or should there be substantial devolution of demand mechanisms in, for example, the form of quasi-markets in which the demand side is revealed by local commissioning agencies such as health authorities or regional offices of the Health Ministry?

### 2.2 Supplier-induced demand

A second characteristic of health care R&D is the dominant role traditionally played in it by researchers themselves. There can be no question that the suppliers of R&D have an irreplaceable practical role in formulating the demand for R&D, for example, by sorting questions into those that are researchable and those that are not, in developing protocols, in commissioning work, and in assessing the quality of what is done. Many may also have a shrewd appreciation of the kind of work that is needed in the health care system, through long acquaintance with its working, through the roles many of them have as board members of hospitals, health authorities and research funders. On the other hand, researchers also have their own research agendas and this provides the potential for supplier-induced demand (Evans, 1974; Labelle *et al*., 1994). Those in universities tend to have a preference for curiosity-driven research, have in mind the priority criteria of their immediate peers in such matters as promotion in universities and hospitals, and most would prefer to set their own standards of excellence to having others thrust standards upon them. Those in industry also have priorities that may not correspond to the public interest. Both are likely always to want ‘more’ regardless of whether ‘more’ would be optimal from any wider social perspective.

Given the chance, therefore, one may expect the research community, as suppliers of R&D, to induce demand whenever they can and to give it a bias towards the particular agendas they have as curious people and people with careers and commercial interests to promote. It therefore becomes important to ensure that the mechanisms for revealing the public demand for R&D, and for meeting it, are not contaminated by factors and interests that are irrelevant in determining the appropriate supply of this public good.

### 2.3 Information asymmetries

A third special characteristic of R&D in health care is that asymmetries are all-pervasive. One – the ability to distinguish between an important question and an important researchable question – has already been mentioned. Managers may be good at the former and researchers bad at it; researchers are usually good at the latter but managers bad. Each possesses specialised information and types of accountability that the other does not. The classic asymmetry in health care arises in the doctor–patient relationship, in which the clinical professional has technical information not usually available to the patient and the patient has deep knowledge of personal and family circumstances which may have clinical and other relevance for personal decision making (Evans, 1974; Arrow, 1985; Dranove and White, 1987; Eisenhardt, 1989; Goddard *et al.*, 2000; McGuire, 2000; Chalkley and Khalil 2005). A principal–agent relationship emerges here, as in other professional exchanges with clients, and it follows that the professionals' information should be well-evidenced and maintained in an up-to-date fashion, so that a trust is developed that is not corroded by irrelevant factors, such as the professional's earning opportunities and the agent can effectively serve the interests of the principal, her client. Institutions are therefore required to support high-quality professional practice with publicly accessible evidence-informed information.

Another important kind of asymmetry is that between commissioners and doers of research. Doers will generally have a better idea of the character of the detailed work they will be doing, and of its quality, than commissioners will. This asymmetry can give rise to inefficiencies arising on both sides. Without mechanisms to overcome the problem, commissioners may commission poor work (and may not even know that the work when it is done is poor); researchers may use the information gap between them and their commissioners to pursue interests that are contrary to those of the commissioners.

### 2.4 Joint production

A fourth special characteristic of R&D in health care is that conditions of joint production frequently apply (Mishan, 1969; Harris, 1977; Culyer *et al*., 1978; Sandler and Culyer, 1982; Ma and McGuire, 1993; Butler, 1995). This is particularly true of research that uses patients. The patient in a research institution is there to receive care funded as a frontline service. The research will often require additional or especially varied treatment packages for patients, which arise solely because of the research in question. The issue then arises: are the costs of treatment costs of research or of patient care? The problem is directly analogous to the classic textbook example of wool and mutton: is the cost of the sheep's fodder the cost of the wool or of the mutton? There is no analytical answer to this question – though the question may be empirically answerable as to the cost of an *additional* kilo of meat or an *additional* kilo of wool.

The solution to this conundrum has to consist in the development of methods of cost accounting that do not distort decisions about either care or research and that do not provide systematic incentives for strategic, self-serving behaviour by the various players in the system (Culyer, 1994). Although direct analytical solutions to this problem may elude us, it ought in principle to be possible to design institutional and behavioural arrangements that produce sensible outcomes. After all, in the case of private goods, the jointness of wool and meat production does not prevent an acceptable solution being reached.

### 2.5 Multiplicity of funding sources

A fifth characteristic is that health-related R&D is usually funded by a multiplicity of sources. These include government departments of health, finance and education, major health care and educational professional bodies, domestic funding agencies for health and education, research councils (medical and social science), the health care system itself, industry and a large number of medical research charities. There may be international bodies with funding partnership offerings and other interests. Each typically has specific objects which differ. For example, some emphasise the ‘science’ end of research, others more applied health services research; others will not support cancer-related R&D, others only cancer-related R&D. The availability of a wide variety of different sources for funding research is a protection for research institutions having a (wise) policy of diversifying their funding sources. However, there is no automatic mechanism through which complementarities between funders can be addressed (even obtaining information about different funders' plans is often difficult) and there are few means of identifying overlaps, duplications and gaps in coverage. This is partly a problem of information production and dissemination (itself a public good) and partly a problem of absent coordinating mechanisms.

### 2.6 Uncertainty of outcome

A sixth characteristic is the fact that all research is beset by uncertainty as to the outcome. The project may not ‘work’. The researchers selected may not be sufficiently skilled to complete their task. On the upside, research projects may also yield useful outcomes that were not anticipated. Again, a mechanism is required that is not excessively risk-averse, nor short-term in the thinking it encourages, and that maximises the chances at reasonable cost of the work achieving its set objectives.

### 2.7 Measurement of outcome

Measurement of the outcome and value of research has challenged analysts since the 1990s (e.g. Eddy, 1989; Drummond *et al*., 1992; Buxton 1993; NHS Executive, 1997; Townsend and Buxton, 1997; Harper *et al*., 1998). This seventh characteristic is that the measurement of outcome – especially of ultimate outcome (benefit to patients) – is hard to characterise and calibrate. It may not always be possible to identify ‘success’, especially when the research in question is very long term or aims at devising new algorithms for (say) planners and managers. Since publicly funded applied R&D is usually funded for basically utilitarian reasons, mechanisms are required to enable judgements about outcomes to be made as best as is possible under the circumstances – making them as near to the ultimate benefit as possible and using intermediate outcomes that are themselves useful and which may be good indicators of likely final benefit.

Ultimately, one expects the value of R&D outcomes to determine (assuming that R&D resources are efficiently allocated) the overall level of resource commitment to health care R&D. There may or may not be a public expenditure target but, in any event, agreeing what it ought to be is unlikely to be easy. It is likely to represent a mere stab in the absence of any reliable evidential basis, for example, as to what essential infrastructure is required to nurture and sustain research capacity or encourage bright researchers to make long-term commitments to the field.

### 2.8 Non-profit institutions

An eighth characteristic of health care R&D is that it is often performed under the aegis of non-profit institutions such as hospitals and universities, whose internal mechanisms for supporting research may be quite primitive (even absent) and which certainly vary enormously from institution to institution. A mechanism is therefore also needed that systematically provides incentives for the effective internal organisation and management of research in institutions.

All R&D systems have to confront these problems if they are to be rationally resolved. Plainly, what is needed is not a counsel of perfection. But practical steps might include creating workable arrangements for bringing together the relevant stakeholders in order to invent a system through which specific answers to the prioritisation needs of the country can be arrived at and which embodies routine, and probably highly participative, advisory meetings of the principal players.

One way of developing an infrastructure is to look at what others have done and see whether other countries' arrangements can be adopted or adapted for one's own purposes. In the 1990s, the system of support for health care R&D in England was becoming manifestly unfit for purpose. The next section provides an account of both of the issues confronting the government at the time and the solutions adopted. Since that time there have been many further developments not covered here, but what was done then proved a useful foundation for all that followed.

## 3. Health care R&D in England: the case study

In the 1990s, two major developments had a dramatic impact on the organisation and character of research in and for the NHS. One was the creation in 1991 of the Research and Development Strategy for the NHS (Department of Health, 1991; Peckham, 1991). The other was a 1994 Task Force Report on supporting R&D in the NHS (Culyer, 1994), which led to a substantial overhaul of the methods by which R&D in and for the NHS was funded at the institutional level. Both these developments grew out of a variety of concerns with the arrangements that preceded them.

### 3.1 R&D as a public good: the NHS R&D Programme

The NHS Research and Development Programme was the direct result of the House of Lords Select Committee on Science and Technology Report (House of Lords Select Committee on Science and Technology, 1984) on priorities in medical research that, despite its title's emphasis on medical research, had advocated the creation of a National Health Research Authority, with a broad brief that included health services research. This was regarded both by the Select Committee and the Government as a Cinderella area – but one of importance for the conduct of public policy. Both felt that the NHS lacked mechanisms for identifying and meeting the NHS's need/demand for R&D and that, while much research ought to continue to be science-led, there had developed an imbalance in the pattern of research taken as a whole. The Select Committee also wanted to see a special health authority established in order to keep R&D at arm's length from the Department of Health and hence protect curiosity-driven research.

The Government's response (Department of Health, 1989) was to integrate the R&D function within the Department of Health, the NHS Executive and the (then) regional health authorities. The new post of Director of Research and Development was created with support staff. The task of the new Director was to advise the NHS Management Executive (as it was then called) on the priorities for NHS R&D, to manage a programme of R&D to meet identified needs (especially the effectiveness and efficiency of health services) and to disseminate R&D results to managers and clinicians in the NHS. The key parts of the House of Lords recommendations and of the Government's response clearly relate to central issues of the publicness of R&D, in particular the development of mechanisms for revealing the NHS's demand and ensuring that resources for R&D were not hijacked by purely academic and clinical interests. The task of identifying R&D as a public good and articulating its value thus fell to the new R&D Programme.

### 3.2 R&D outcomes and their valuation: operating principles of the R&D strategy

By 1993, the main elements of the NHS R&D Strategy had been established. Its main objective was to contribute to the health and well-being of the population through the production and application of relevant and high-quality research, and it had six structural features:

- national and regional infrastructures for identifying and prioritising NHS R&D requirements;
- the initiation of major programmes of work on priorities identified by the Central R&D Committee as being important to the NHS;
- a quasi-market mechanism for commissioning R&D via contracts between the Department of Health and research institutions;
- the development of strategic alliances with other health research funders;
- a dissemination strategy;
- a strategy for research training and career development.

The Director of R&D had established a Central R&D Committee, with representatives from the research community, the NHS and (later) consumers of NHS services. Regional programmes attuned to the perceived R&D needs of the regions had been developed from the previous regional arrangements, under new Regional Directors of R&D. The central programme had two basic elements: a set of national priority areas, each time-limited, on a dozen or so topics (such as mental health, cardiovascular disease and stroke, and cancer) and a Standing Group on Health Technology Assessment, which was, as its name implied, a permanent group operating a programme that, more than any other, directly addressed the central concerns of effectiveness and cost-effectiveness. The Standing Group was to be an engine for generating major culture change in the NHS by promoting a more critically aware pattern of professional service delivery based, wherever possible, on reliable evidence about ‘what worked’ and at what cost.

### 3.3 Asymmetry of information

To begin the task of disseminating this work and other relevant research, the Cochrane Centre at Oxford and the NHS Centre for Reviews and Dissemination at York were established. A National Project Register, containing information on all R&D of interest to the NHS was also set up. The development of mechanisms addressing the training implications of the R&D programme and the capacity requirements of the research community if the needs identified by the programme were to be met were somewhat postponed (Baker, 1998).

The Central Committee had had several discussions on the definition of R&D but, apart from determining that development ought not to include the service development work conducted by hospitals and others as a part of their normal operating processes, agreed to go no further than define some pragmatic criteria for funding NHS R&D. These criteria were that funded work should be designed to: provide new knowledge considered necessary to improve the performance of the NHS in enhancing the nation's health (a dimension of publicness but locating priority decisions with commissioners, hence reducing the influence of supplier-induced demand); produce results of value to those in the NHS facing similar problems outside the particular locality or context of the project (publicness), the judgement of ‘value’ or research ‘outcome’ becoming effectively a responsibility of commissioners and being determined through deliberative processes; follow a clear, well-defined protocol (quality control); have clearly defined arrangements for project management (quality control); have the clear intention to report findings so that they are open to critical appraisal and generally accessible (publicness and quality control) (Department of Health, 1993). It was also agreed early on that final decisions about priorities and commissioning could not be substantially devolved (e.g. to local health authorities). This was partly on grounds of information asymmetry – many local authorities and local commissioning groups judged to be lacking abilities to act in a sufficiently well-informed way as to be effective agents, partly because of the limited national availability of skills, their highly unequal geographical spread, and the likely demands that would be placed on the people possessing them, and partly because NHS R&D was seen as a public good whose benefits flowed primarily to the entire country.

The Standing Group on Health Technology (Department of Health, 1997a, 1997b, 1997c, 1997d) supervised the work of six advisory panels, covering the acute sector, diagnostics and imaging, pharmaceuticals, population screening, primary and community care, and methodology. Each panel had academic and NHS membership, observers from the Medical Research Council, Department of Health and the NHS Executive, and each was to acquire ‘consumer’ members (which were trialled successfully in the Methodology Panel!). The potential for consumer participation was discussed in Department of Health (1998) but implemented only cautiously and later though it did develop into a core element of the R&D programme over the years (Department of Health, 1998).

### 3.4 Minimising SID

The arrangements sought to minimise SID while ensuring that commissioning was informed by expert research opinion. Each panel conducted an annual consultation exercise within the Department of Health, the NHS Executive, NHS management, NHS clinicians (including nurses and professions allied to medicine) and the academic community to elicit proposals (1800 in 1997). They were then further sifted by the Secretariat in discussion with Panel Chairs, mostly medical academics, into a total of about 100 topics. These researchable proposals were then further developed, through an iterative process, into ‘vignettes’ by the Secretariat describing the nature of the work required, its likely results, its likely benefits to the NHS and its likely costs. These were then reviewed by each Panel, scored into three priority categories. At this stage, the possibility of external or joint support was explored (e.g. with the Medical Research Council) and bids for the work were then sought through an open public bidding process.

### 3.5 Fragmentation

The other major innovation resulted from the acceptance by the Government of the recommendations of the Department of Health's 1994 Task Force Report (Culyer, 1994), which addressed the question of how best to fund the infrastructure for research in institutions. In 1993, the principal sources by which the R&D of teaching hospitals was funded were diverse and problematic (fragmentation). The largest single source of specific project funding was industry – though there was considerable variance in the quality of this work. There was also the Department of Health's Service Increment for Teaching and Research (SIFTR) – the main source of R&D infrastructure support for teaching hospitals but oddly a function of undergraduate student numbers rather than research students or research. This was the principal means through which the Secretary of State's statutory obligation to support research in the NHS was fulfilled, a task which had usually been interpreted as an obligation to support Medical Research Council projects in the NHS, and was based on earlier attempts to estimate the cost of teaching in hospitals (Culyer *et al*., 1978); non-SIFTR – the appropriately named support for the few teaching hospitals with no medical undergraduates; the specially negotiated annual research support for the London postgraduate hospitals; some specifically ‘tasked’(earmarked) money for academic general practice research; and the ‘own account’ research undertaken by hospitals from within the resources provided for them for patient care – research whose quality was often unknown and whose total size of funding was also quite unknown.

This system had grown in an *ad hoc* fashion, mostly as a consequence of problems as these arose. It was medically dominated; it was institution-focussed (to the neglect of community-based NHS practice); it was largely arbitrary (e.g. the tie-in of funding to undergraduate numbers); it could not be used as an instrument for enhancing the quality or encouraging a focus on the needs of the NHS; there were complaints from researchers in hospitals that the funding was not actually supporting research; it was impossible to account for it – or to hold anyone to account for it; the quality of much of the work it was supporting was alleged to be poor; and it amounted, for the most part, to a general subsidy to institutions (Department of Health, 1997a, 1997b, 1997c, 1997d). The subsidy had virtually no strings attached and, in particular, no means for assessing (or encouraging) quality. Moreover, SIFTR, non-SIFTR and the special arrangements for the London postgraduate hospitals were all very institutionally focussed streams of funding. Not only did they exclude all community-based R&D, which was odd since the community was becoming increasingly the location of health care delivery, but they provided no means of support for partnerships between institutions. For a comprehensive account of the perceived faults of the previous systems, see Culyer (1995).

Following the introduction of the internal market for patient services (National Health Service and Community Care Act, 1990), a further problem had arisen for secondary care providers who engaged in ‘own account’ non-commercially funded research. This was that, since their prices incorporated the costs of such research, they were increasingly at a competitive disadvantage as health authorities and fund-holding GPs sought the least cost packages from their service contracts. This led to fears of a substantial squeeze on R&D and that ‘own account’ research funding would be driven out. The research community in such institutions, some very distinguished, had made very public its concerns at the one-sided way in which the newly established market was prejudicing R&D in the absence of any corresponding quasi-market structure for R&D. However, and conversely, there were also indications that institutions whose research was relatively well supported were subsidising patient care prices. The mechanisms then extant afforded little protection against either hazard.

What replaced this miscellaneous assemblage of *ad hoc* measures was radical (Department of Health, 1996a, 1996b, 1997a, 1997b). First, the general source of all funding for both the R&D programme itself and the infrastructure support became a Levy (along with several others such as that for Non-Medical Education and Training) on local health authorities. This enabled research-intensive hospitals and other clinical practices to bid competitively for R&D resources and meant that the R&D costs were effectively removed from the prices for health care sought by providers from purchasers.

Second, a new National Forum was created at which all the principal players in the R&D system (the universities, the High Education Funding Council for England, the NHS Executive, the relevant research councils, the medical charities and industry) were represented and which could address common issues on both the demand and the supply sides and offer advice both to the NHS Director of R&D and their own constituencies.

Third, a new bidding system was introduced to replace SIFTR, non-SIFTR and the special arrangements for the London postgraduate hospitals. Not only could institutions bid for this stream of funding but also consortia – the latter opportunity being particularly directed at community-based service providers with R&D capability to enable them to collaborate with other similar groups to achieve scale economies and with established centres of excellence to enhance quality. Two types of time-limited contract were introduced, called Portfolio and Task-Linked, the first of which was designed for large institutions with predictable needs for Medical Research Council and other non-commercial research support and which had the general form of a block grant with relatively little specific monitoring of specific components or outcomes, thus minimising contracting costs; the second format was designed for institutions or consortia of community-based practices whose quality and track record were more difficult to ascertain and whose R&D objectives were more tightly negotiated. A set of 10 criteria was established (Department of Health, 1996b) for evaluating bids. These related to expected flows of non-commercial external support, the quality of research management and the relevance of the bidders' plans to the needs of the NHS.

To manage these radical changes, an elaborate system of regional evaluation followed by central arbitration to ensure consistency in the application of the criteria and to make marginal adjustments in the light of expected productivity was developed. The Task Force had recommended that the changes be introduced without serious destabilisation of existing recipients of previous streams of funding. Final allocations were agreed with NHS Regional Directors. The mechanism thus placed the articulation and quantification of demand clearly in the hands of the NHS Executive on behalf of the NHS, created a national competition between R&D suppliers for the resources, and instituted a set of incentives for institutions to manage their R&D work more efficiently.

### 3.6 Dealing with jointness

The introduction of the new system replacing SIFTR, etc., had been preceded by an important accounting exercise in which all recipients had been asked to declare all their non-commercial sources of R&D income and to account as far as possible (and with some fairly heroic assumptions in the case of joint costs) for existing R&D expenditures. This exercise was successfully accomplished with a minimum of (detected) gamesmanship and served as the benchmark against which to judge the subsequent movement of funds. The exercise required bidders both to set out what they intend to achieve, with measurable outcomes, and their own estimates of what it would cost. The new system thus attempted to establish a well-designed quasi-market, with the ultimate judgements about the value of public good outputs being made by the Director of R&D advised by the Central R&D Committee, the reasonableness of the costs being judged by central and regional experts, and plenty of scope for individual institutions to display their own initiative and set their own priorities in the light of what they know to be the broad priorities set by ministers. In this way, the system sought to make reasonable judgements about the issues of jointness with, so far as possible, costs of patient care falling on institutions' health care budgets and costs of R&D falling on the R&D budget.

Overall funding for medical and health-related research in England in the mid-1990s amounted to about £3299 million per year, roughly 5.4% of the budget of the NHS in 1994/5. Of this, industry (primarily pharmaceuticals) contributed about £2000 million, research charities £340 million, the Medical Research Council £278 million and the Higher Education Funding Council for England £190 million (medical schools). The Department of Health and the NHS contributed £491 million, of which £65 million came from the Department of Health and £426 million from the NHS.

**Figure 1.**
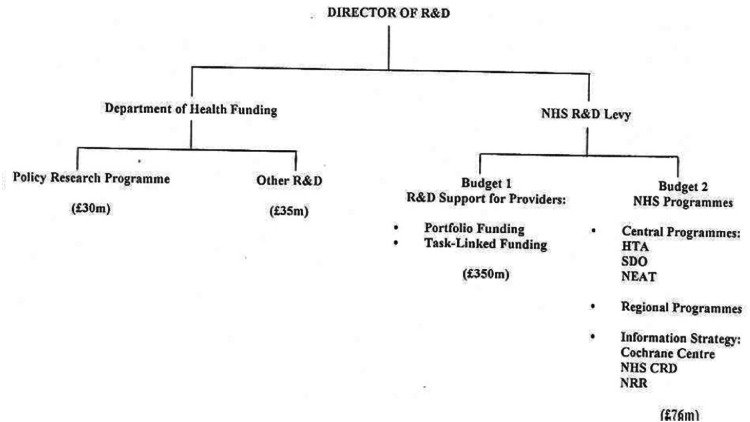
R&D funding streams in England 1994 (Culyer, 1994).

The new arrangements for NHS R&D (both NHS-funded R&D and NHS support for the non-commercial R&D funded by others) are shown in Figure 1. R&D in the NHS was now comprehensively managed by the Director of R&D on the advice of the Central R&D Committee. There were two broad divisions into which the work was divided: that funded directly by the Department of Health and that funded out of the R&D Levy.

On the left of the figure, directly funded R&D fell into two categories. One was work commissioned by the Department of Health as a part of its policy development activity (usually determined by ministerial priorities and directly related to political judgements of the public good need for relevant R&D). This was the Policy Research Programme, which includes social care research as well as health and health services research. Second from the left were a miscellany of other centrally funded R&D activities such as the specific programme managed by the High Security Psychiatric Hospitals Commissioning Board.

On the right were the activities supported by the R&D Levy. ‘Budget 1’ was the new form of support for NHS providers – the new single stream that replaced SIFTR, non-SIFTR, etc., which was available through the processes described in the form either of Portfolio or Task-Linked Funding. ‘Budget 2’ funded the NHS R&D Programme, which now consisted of three central programmes where the scope of publicness was NHS-wide: the Health Technology Assessment Programme under the Standing Group, two new programmes also with standing groups in Service Delivery and Organisation, and New and Emerging Applications of Technology, the Regional Programmes of support for work that was also national in its publicness but of lower general priority while being of particular regional concern, and the Information Strategy consisting of the Cochrane Centre, the NHS Centre for Reviews and Dissemination, and the National Research Register, formerly the National Project Register.

### 3.7 Non-profit institutions

The essential character of a public good like R&D is defined in terms of its demand-side character. The publicness of R&D is, however, compounded by supply-side matters which policy needs also to address if the level of activity is to be even approximately optimised. A major characteristic of health-related R&D is that it is located principally in universities and hospitals or clinical practices associated with universities. Universities are independent non-profit private organisations in the UK (though heavily dominated by their principal funder – the state) that pursue agendas that are their own and, in the case of research, are largely determined by the preferences and values of researchers themselves. Unfortunately, the economic (or any other behavioural) theory of institutions such as universities is poorly developed (but see Newhouse, 1970; Holtmann, 1988; Sperling, 2008) and offers little in the way of guidance as to what might be expected if their environment were manipulated. Academic freedom is jealously guarded and, even when research projects are commissioned by external bodies, researchers typically seek to promote their own priorities as far as possible on the backs of such sponsorship (SID). This plainly creates tensions that require appropriate trading off in the R&D commissioning process and, for the most part, the Department of Health and the NHS R&D Programme have usually seen their own interest as also being served by helping to ensure the continuation of a vibrant research community that is not entirely and exclusively driven by the needs (short or long term) of the NHS.

More difficult was the issue of the efficiency with which universities managed their research activity. Most universities adopted a ‘bottom-up’ approach in which the research priorities of the institutions were derived from the preferences of the researchers themselves and the resource and support structures provided are those preferred by the same group. Until the advent of the Research Assessment Exercises of the Higher Education Funding Councils in 1986, however, there was typically little thought given to the management and support of research within institutions, little sharing of good practice, and remarkably little attention given to the career interests of an increasing proportion of staff who were funded by external research support grants with a limited life (most, for example, took the arbitrary view that the life of the employment contract should be the same as the life of the support grant). Research management and support in hospitals was substantially worse and many claiming a serious commitment to R&D lacked a senior manager and support staff to develop strategies through which it could be made to flourish. The culture change required began to be significantly achieved only after the creation of the RAEs and their successors and the advent of NICE in 1999, now the National Institute for Health and Care Excellence. On the later history of R&D in the NHS, see Hanney *et al*. (2010), Atkinson (2019).

## 4. Conclusions

This paper has sought to analyse R&D as a public good, whose demand ought to be revealed by those with the technical competencies to do so (e.g. ability to distinguish the researchable from the un-researchable) and those with political legitimacy and accountability who can articulate a need for a knowledge evidence base regarding ‘what works?’, ‘how well does it work and for whom?’, and ‘at what cost relative to the alternatives?’. It has identified seven other characteristics of R&D that further compound the problems in securing appropriate and timely delivery: the fact that supplier-induced demand may distort the optimal pattern; the presence of important information asymmetries; the jointness in production between research, education and patient service delivery; the multiplicity of research funders; the inherent riskiness of research; the subtlety of the output of research and its lack of amenability to sensible measurement and valuation; and the fact that it is typically produced (even when industry-funded) in private institutions (as universities are in the UK). We offer these eight characteristics as a possibly useful starting point for those seeking to develop a health R&D strategy in their own country. They are intended to provide a broad framework for thinking about the most fundamental issues that are likely to arise in any discussion of public policy health care R&D: a framework for thought, not a substitute for it. The example we give demonstrates that it is possible to create order and better public control even when the starting point is chaotic and ridden with historical anomalies and special interests, and where there is much uncertainty about the ‘what works?’ questions, and to do so with a reasonably smooth transition from the old to the new.

We describe and discuss the arrangements for supporting R&D in the NHS in the 1990s to illustrate the ways in which one jurisdiction addressed these problems. This experience is not intended to be a model. The ‘what works?’, ‘how well does it work and for whom’, and ‘at what cost relative to the alternatives?’ are questions we have not here sought to apply to the experience of the NHS in the 1990s, though they are certainly worthy of attention. There is little literature available and what is available is not directly relevant. The interested reader might begin with Buxton's work (Brunel University London) and RAND's commission (RAND Corporation n.d.).

The circumstances leading to the changes described were specific to the NHS as were the traditions and customary practices. Each jurisdiction will have its own hallmarks. At the time, the UK as a whole was also going through a period of active promotion of quasi-market structures in the public sector as a whole, intended to provide suitable incentives for achieving better outcomes. Nonetheless, it seems possible that there are sufficient parallels between this relatively wealthy country, with a long-established health care system having strong features of comprehensiveness and universality, for local adaptations to be selected and worked up into policy strategies elsewhere. In addition, there is the potential, as one of us has argued elsewhere (Chalkidou, 2019), for using the significant resources committed to research from the ODA budget to support other countries in setting up their own health system research institutions and national health research agendas as they journey towards UHC.
